# Nanocomposite Fiber Based on Natural Material for Water Disinfection under Visible Light Irradiation

**DOI:** 10.3390/nano10061192

**Published:** 2020-06-18

**Authors:** Faissal Aziz, Mounir El Achaby, Khalid Aziz, Naaila Ouazzani, Laila Mandi, Mohamed Nawfal Ghazzal

**Affiliations:** 1 Laboratory of Water, Biodiversity & Climate Change, Semlalia Faculty of Sciences, Cadi Ayyad University, Marrakech 4000, Morocco; ouazzani@uca.ma (N.O.); mandi@uca.ma (L.M.); 2 National Centre for Research and Study on Water and Energy (CNEREE), Cadi Ayyad University, Marrakech 40000, Morocco; 3 Materials Science and Nano-engineering (MSN) Department, Mohammed VI Polytechnic University (UM 6 P), Benguerir 43150, Morocco; mounir.elachaby@um6p.ma; 4 Materials, Catalysis and Valorization of Natural Resources, Faculty of Sciences, Ibn Zohr University, BP 8106, Agadir 80000, Morocco; khalid.aziz@edu.uiz.ac.ma; 5 Institut de Chimie Physique, UMR8000 CNRS, Université Paris-Saclay, 91405 Orsay, France

**Keywords:** photocatalysis, methylene, goethite, nanocomposite fiber, bacteria, visible-light

## Abstract

In the last decade, pathogenic bacteria and organic micropollutants have become a major issue in the water purification process. Heterogeneous photocatalysis is a low-cost and an ecofriendly process, which provides a sustainable solution for water treatment and its utilization in rural areas. In this context, we studied the generation and the surface engineering of polyacrylonitrile (PAN)/goethite composite nanofibers for photocatalytic water remediation under visible-light illumination. The photocatalytic activity was evaluated for dye (methylene blue) degradation and bacteria inactivation, as contaminant models, of the composite nanofibers. The PAN/goethite nanofibers were elaborated by an electrospinning technique, and the morphology and the composition, before and after spin coating, were investigated by Scanning Electron Microscopy (SEM) and Energy Dispersive X-Ray (EDX). The results showed partially intercalated structures of the PAN/goethite Composite-nano-fiber (CNF) were identified by SEM and EDX analysis. The composite nanofibers exhibited high photoefficiency upon dye bleaching (only 10% left after 5 h of illumination) and bacterial deactivation *Escherichia coli* and *Clostridium perfringens* (4.4- and 3.5-fold, respectively, in less than 5 h). The steadiness and pliancy of the generated nanofibers provide a promising application in the continuous flow system.

## 1. Introduction 

Daily access to safe drinking water is an essential priority to maintain the health and quality of life. However, while this seems a mere formality in some parts of the world, the reality is that millions of people still lack access to safe drinking water. The lack of safe drinking water is identified as one of the major causes of high-risk waterborne diseases, such as cholera, typhoid fever, hepatitis A, dysentery, and diarrhea [1]. In 2013, diarrhea was the cause of the 1600 daily deaths among children under five years old worldwide [2].

Most developing countries are experiencing a scarcity of freshwater resources accompanied by poorly developed infrastructure, especially in rural and suburban areas that rely mainly on archaic resources and conventional techniques to supply drinking water. These resources and techniques contribute to the breakthrough of epidemiological disease due to the high risk of water contamination, particularly by pathogenic bacteria [3,4]. In these areas, chlorine disinfection is often the only treatment available for drinking water. However, chlorine disinfection has not always been successfully implemented due to financial problems resulting in intermittent disinfection at the correct dose, poor operation, and poor maintenance. In addition, in the presence of natural organic molecules (NOMs), chlorination leads to the formation of carcinogenic disinfection by-products (DBPs), trihalomethanes (THMs) [5,6]. In this context, there is an urgent need to provide an innovative, low-cost, and efficient technology for water decontamination. The decontamination process should be easy to set-up and require very little maintenance. 

Photocatalysis is one of the promising techniques for water disinfection for both drinking water and wastewater [7,8]. In this line, photocatalytic degradation is approved as an efficient and economic technique in treating aqueous media with harmful bacteria and organic pollutants [9,10,11,12]. The photocatalysts, i.e., the motor of the photocatalytic processes, with high activities include titanium oxide, zinc oxide, and iron oxides [13,14,15,16].

Iron oxides are among earth-abundant materials [17,18], such as goethite (R-FeOOH) and hematite (R-Fe_2_O_3_), which are common in soils and sediments. α-FeO_3_, γ-Fe_2_O_3_, α-FeOOH, β-FeOOH, and γ-FeOOH show good photoactivity for organic pollutant degradation and water treatment [19,20]. However, iron oxides as photocatalysts are less investigated compared to TiO_2_ or ZnO. Iron oxide has the advantage of absorbing visible light, which is inaccessible to the most studied TiO_2_ photocatalysts.

Compared to many conventional materials such as TiO_2_, ZnO, etc., α-Fe_2_O_3_ has an advantage in the photocatalytic process especially under solar energy due to its lower bandgap ~2.2 eV value. This advantage is related to iron oxide capacity to absorb a large portion of the visible solar spectrum (absorbance edge ~600 nm). Moreover, it is considered a promising material for photocatalytic water treatment due to its good chemical stability in an aqueous medium, low cost, abundance, and non-toxic nature. However, Fe_2_O_3_ usage is limited by some disadvantages such as a higher e-h recombination effect, low diffusion length and valence band (VB) positioning (VB is positive with respect to H^+^/H_2_ potential) [21].

Iron oxides nanoparticles are synthetized using different techniques, including template-assisted synthesis [22,23], biological synthesis [24], hydrothermal synthesis [25,26], vapor-solid growth techniques [27,28], and the sol–gel process [29,30]. These techniques require specialized equipment, operate at high temperature, and lead in some cases to the production of dangerous by-products during the synthesis process [26]. Electrospinning is another emerging method for material synthesis, which enables us to obtain polymer fibers with diameters ranging from the nano- to the microscale [30]. Nanofibers can provide a high specific surface area and the ability to stretch, bend, and roll up nanoparticle sheets when introduced as part of a multilayer fibrous form [31,32,33].

In this study, we propose the elaboration of a natural-based photoactive membrane by the electrospinning process. The process enables the incorporation of goethite nanoparticles previously extracted from natural materials in a polymer’s nanofiber using a one-step electrospinning protocol. After morphological and structural characterization of the biobased nanocomposite, we evaluated its photocatalytic activity for methylene blue degradation and inactivation of *Escherichia coli* and spores of *clostridium perfringens* (viability of vegetative cells) under visible light illumination.

## 2. Experimental Section

### 2.1. Composite Nano-Fiber (CNF) Generation by the Electrospinning Process

Raw goethite rocks were collected from a Moroccan abandoned mine in the west of the country (Taouz area, Errachidia Province). The nanoparticles were obtained by high-energy ball milling of the goethite rock. The metallic iron rocks were wet milled in a planetary ball mill equipped with stainless steel vials using iron balls for 5 h. The ball-to-powder mass ratio was 40:1, and the rotation speed was fixed at 450 rpm.

A spun solution was prepared by mixing 2% (wt) of goethite nanoparticles, already prepared, and dimethylformamide (DMF) solution. The solution was stirred for 24–30 h at room temperature and then sonicated for 2 h to obtain a homogeneous solution. Then, 10% (wt) of polyacrylonitrile (PAN) polymer was added to the homogeneous mixture and used for the electrospinning process (Invenso NS1 NanoSpinner, Istanbul, Turkey). The electrospinning was performed using a homemade setup, which consisted of a 5 mL glass syringe where the needle diameter was 0.51 mm. The spun solution was pumped mechanically, and the ground electrode (aluminum sheet) was connected to a high voltage power supply. The distance between the needle’s tip and the ground electrode “collector” was 10 cm, the applied voltage was 12 kV, and the flow rate of the solution was 0.4 mL.h^−1^.

### 2.2. Material Characterization

The structural property of the generated nanoparticles was characterized by X-ray diffraction (XRD); the patterns were recorded using Bragg’s configuration range of 0–90° using Cu Kα radiation (40 kV, 35 mA, λ = 1.5418 Å, 2θ), and the goniometer speed was 2°/min. The goethite nanoparticles were imaged with a scanning electron microscope, Zeiss Ultra 55 SEM instrument (Oberkochen, Germany), equipped with an energy-dispersive X-ray (EDX) detector operated at an accelerating voltage of 10–20 keV.

Images of the generated nanoparticles were taken using a High-resolution transmission electron microscope (HR-TEM, JEOL2100 FEG-TEM) at a 200 kV accelerating voltage.

Characterization of the generated goethite CNF: For visualization of the nanoparticles on the nano-composite membranes, a Zeiss Ultra 55 Scanning electron microscope (SEM) was used at an accelerating voltage of 5–10 keV, equipped with an energy-dispersive X-ray (EDX) detector. The nanofiber images were processed by ImageJ analysis software (Stockholm, Sweden).

### 2.3. Evaluation of the Composite Nanofibers’ Photocatalytic Activity

#### 2.3.1. Methylene Blue Bleaching Test

Methylene blue (MB) bleaching experiments were performed in both batch and continuous modes. In batch mode, 0.5 g of the CNF was mixed with 100 mL of the MB solution (10 ppm) in a quartz photoreactor (40 × 15 × 3 cm) under stirring at room temperature during 30 min in the dark before illumination. Then, the photoreactor containing the photocatalyst and the MB dye was placed at 6 cm vertical distance from a Philips lighting 36 W T8 fluorescent tube (270.8 kLux), which was used as the visible light source.

**Figure 1:**
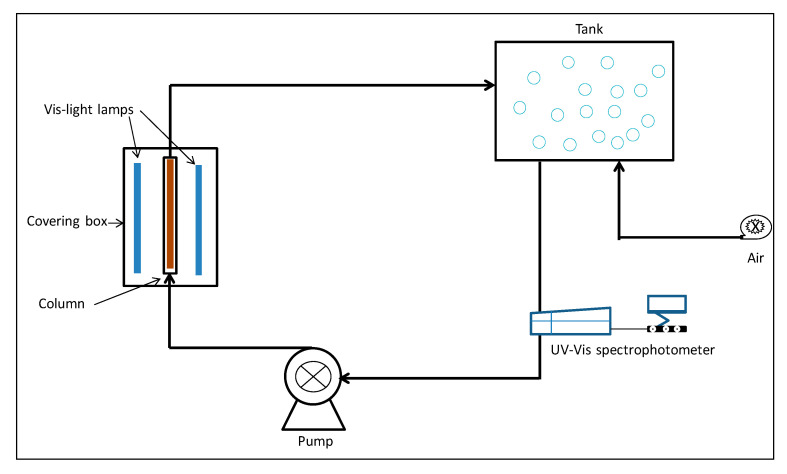
Schematic diagram of the photocatalytic reactor continuous flow system.

Under the same conditions, control experiments were conducted; we tested the photocatalytic activity of the generated goethite nanoparticles and the PAN nanofiber, taking into consideration the same ratio (wt/v). In the continuous circulation mode, a quartz photoreactor column containing the CNF material (25 cm height and 2 cm diameter) was connected to a tank (a flat bottom bottle of 500 mL capacity), initially filled with 100 mL dye solution, and to a UV-Vis/NIR spectrophotometer (LAMBDA 750, Perkin Elmer, Bridgeport, CT, USA) equipped with a 1 cm-length quartz flow cell. The continuous flow was carried out by means a peristaltic pump at a constant flow speed of 5 mL.min^−1^ (Figure 1). The change in the absorption spectra of the MB was monitored at λ = 660 nm. Before starting the illumination, the solution was put into circulation for 30 min in the dark to reach the adsorption equilibrium. These experiments were repeated without using the nanofiber composite as a control to assess the photolysis of MB dye.

After the adsorption process, the tested membranes were dried and the K/S values were measured using the Xrite-8400 spectrophotometer before and after visible light irradiation. In order to evaluate the adsorption of the dye at the nanofiber composite surface, the relative color strength (K/S) and staining of the membrane were determined according to the Kubelka–Munk Equation (1).

(1) $$\frac{K}{S}=\frac{1-R^{2}}{2R}$$

where K is the absorption coefficient, S is the scattering coefficient, and *R* is the fraction reflectance (value from 0 to 1) of the dyed substrate at the wavelength of minimum reflectance.

The photocatalytic activity was quantitively determined by the bleaching ratio of MB using Equation (2):

(2) $$\mathrm{Degradation}\;\mathrm{efficiency}\;(\%)\;=\frac{C_{0}-C_{i}}{C_{0}}$$

where *C*_0_ is the initial concentration (mg.l^−1^) and *C_i_* is the concentration (mg.l^−1^) at variable illumination times (h).

The photocatalytic reaction rate was assumed to follow the Langmuir–Hinshelwood model [34] that is described by pseudo-first order model [35] in Equation (3):

(3) $$\ln\;\left(\frac{{}_{C_{0}}}{C_{t}}\right)=kt$$

where *k* is the reaction rate constant, *C*_0_ is the initial concentration, and *C_t_* is the concentration at variable illumination time *t*.

To assess the recyclability of the CNF, the process was repeated for up to five cycles. The nanomembrane was repeatedly used without any treatment; after each photocatalytic process, the reactor light was turned off, and the tank was filled once again from the MB stock solution. After half an hour, the reactor was irradiated with visible light, and the MB removal efficiency was computed after each cycle.

#### 2.3.2. Bacterial Inactivation

Two types of bacteria were used as models in this study, *Escherichia coli* (ATCC 8739) and *Clostridium perfringens* (NCTC 8239) from the Laboratory of Microbiology and Biotechnology Type Culture Collection, Marrakech, Morocco; *Escherichia coli* (*E. coli*) as a conventional bacterial indicator and *Clostridium perfringens a* known as a resistant sulphite-reducing anaerobic (SRA) bacterium [3]. *E. coli* and *Clostridium perfringens* are non-pathogenic gram-negative and gram-positive bacteria, respectively, and are well-known models for laboratory experiments. Stock cultures of *Clostridium perfringens* (3.5 log unit CFU/100 mL) and *E. coli* (4.5 log unit CFU/100 mL) were kept in a biological refrigeration room at 4 °C. During the disinfection experiment, a sample was taken each 30 min for analysis.

*Clostridium perfringens* counting was performed on SPS agar medium (Sulfite Polymyxin Sulfadiazine and Cysteine); the mother solution was heat-treated at 80 °C for 10 min, and the samples were incubated for 48 h in anaerobic jars at 37 °C, according to Moroccan Standard 08.0.125 [36]. *E. coli* counting was achieved on Soybean-Casein Digest agar medium; Petri dishes were incubated at 37 °C for 24 h according to Moroccan Standard 08.0.124 [36].

## 3. Results and Discussion

### 3.1. Nanoparticle and Nanocomposite Membrane Characterization

#### 3.1.1. Characterization of Goethite Nanoparticles

**Figure 2:**
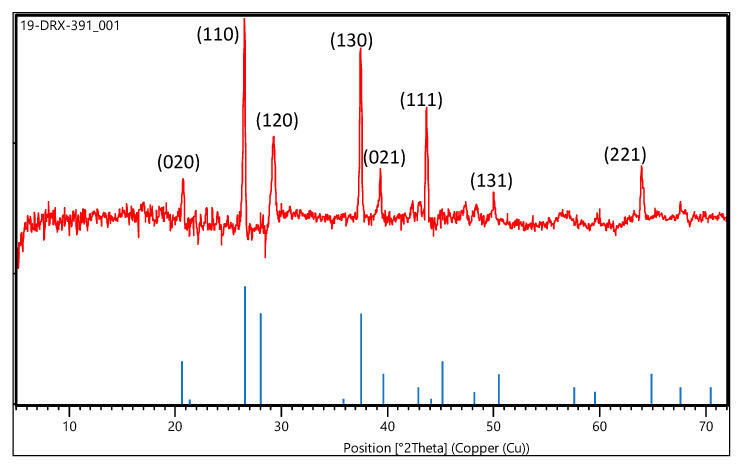
XRD patterns of the generated nanoparticles.

The XRD patterns are shown in Figure 2. The particle structure consisted of hexagonal crystals. The peak height can be an indicator of relative crystallinity; sharper and longer peaks indicate higher crystallinity. Furthermore, the XRD pattern of the nanoparticle sample revealed the presence of 2-line ferrihydrite (Fe_5_ HO_8_·4H_2_O), which was identified by its reflection at approximately 28° and 38° in 2 Theta. The XRD patterns of the generated materials were compared with available diffraction data from the ICDD (International Centre for Diffraction Data) and other references [37,38]. The analysis sample had an α-FeOOH structure, with peaks of low intensity and a broad peak indicating fine particles. Moreover, a small peak at 22° attributed to the α-Fe_2_O_3_ (hematite) structure was noted, even though the content was only a few percent.

**Figure 3:**
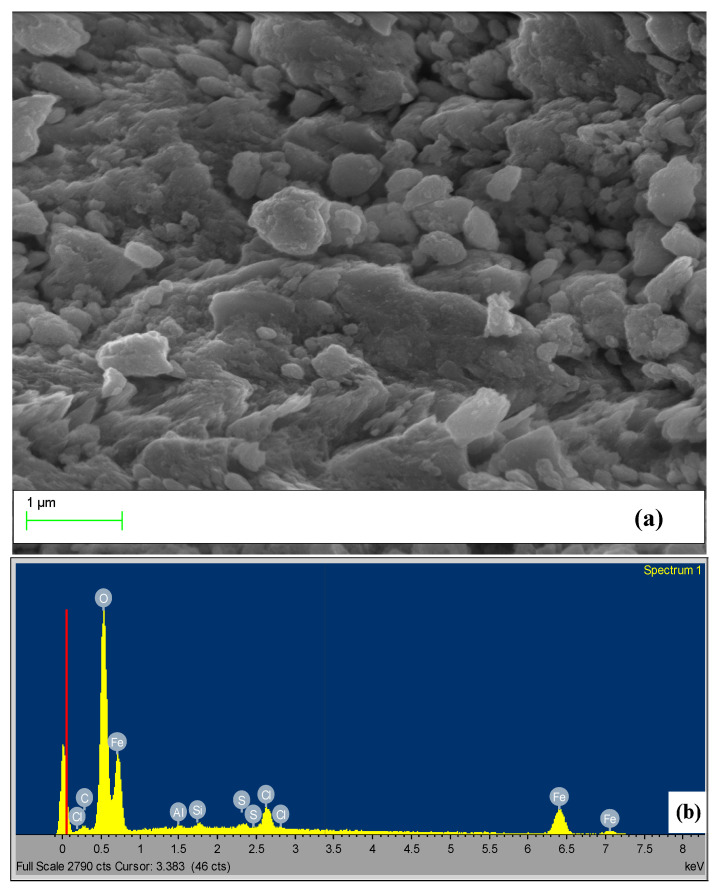
SEM analysis of the goethite nanoparticles: (**a**) SEM image, (**b**) EDX elemental mapping.

**Figure 4:**
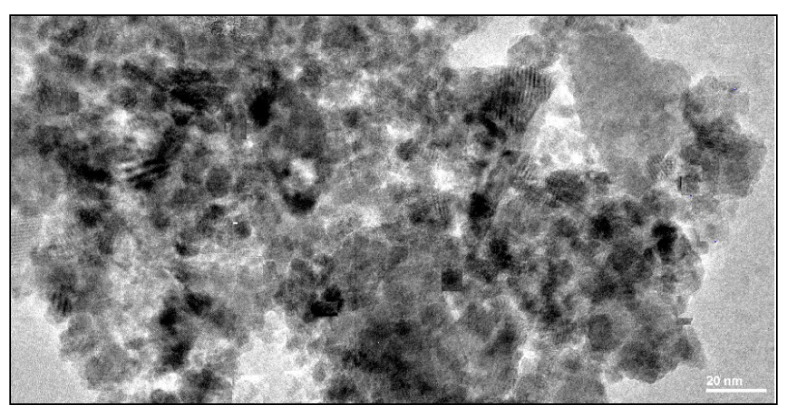
TEM images of the generated nanoparticles.

In line with these results are the EDX outcomes (Figure 3 and Table 1) that show these generated nanoparticles were rich in iron (Fe), with an average around 58%, which gave these particles high photo reactivity. The TEM image (Figure 4) shows that the sample consisted of agglomerates of particles with an average grain size around 6 nm, which indicates ideal nanocrystals since if any aggregates are formed, will disrupt the PAN polymer.

#### 3.1.2. Characterization of Goethite Nanocomposite Fiber

**Figure 5:**
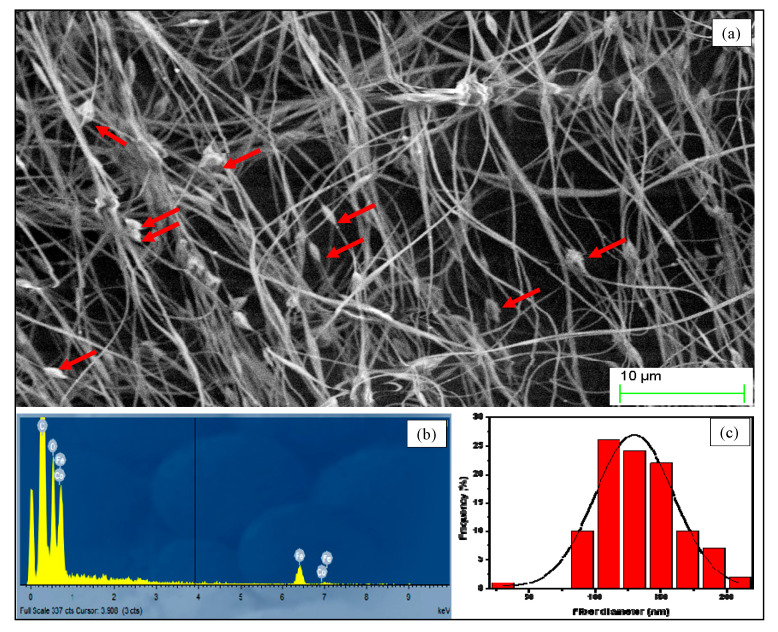
SEM analysis of the nanocomposite fiber: SEM image (**a**) EDX elemental mapping (**b**) nanofiber size distribution (**c**).

The morphology of the nanocomposite was studied by SEM, and the result is presented in Figure 5a. The image shows homogenous fibers in terms of diameter that ranged from 100–150 nm. The surface of CNF did not show any serious cracks or degradation. The goethite nanoparticles were grafted on the surface of the fiber, forming a nanocomposite, confirming the success of the synthetic spinning method. The nanoparticles were homogenously dispersed over all the nanofibers with a size of 133 ± 15 nm, forming a pearl-like structure. It could be claimed that electrospinning and functional nanofibers can still be made in the presence of large aggregates. Due to the aggregation of the nanoparticles, composite beads formed on the nanofiber mat and remained active because the aggregates were confined to large pearls. Furthermore, the surface investigations of CNF based on the SEM images in Figure 5 show that all goethite particles were exposed at the surface of the fiber, which confirms the formation of an exfoliated-intercalated structure. The synthesized nanofiber showed considerable stability in various buffers and at various pH values, and the nanoparticles were found to have potential for bio-application. The nanocomposite was further analyzed by EDX, and the result is presented in Figure 5b and Table 2. The results indicate the presence of Fe, O, C, Al and Si elements and the principal components goethite and natural fibers.

### 3.2. Methylene Blue Removal

#### 3.2.1. Control Experiments

**Figure 6:**
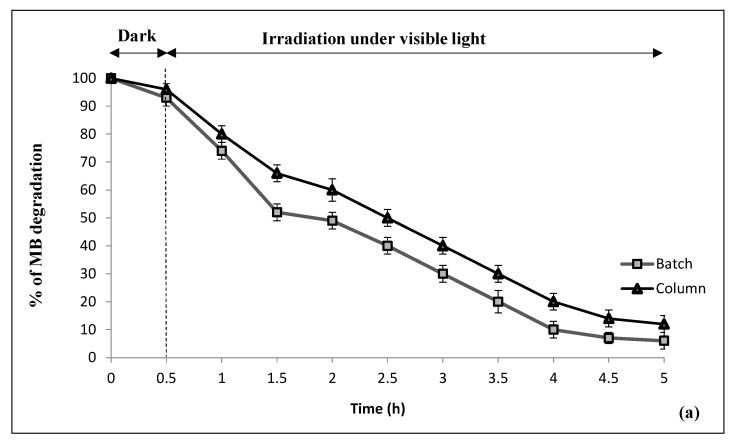

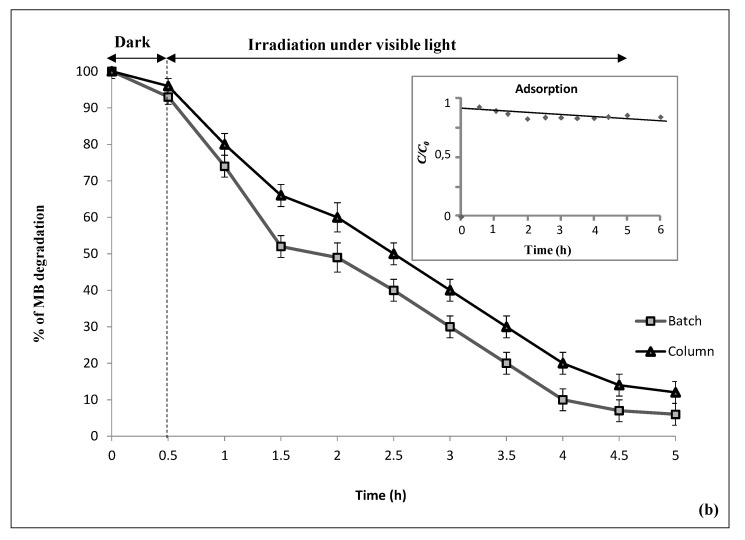
Kinetics of methylene blue degradation in the dark and under visible light irradiation by (**a**) the goethite nanoparticles and PAN nanofiber; (**b**) the natural nanocomposite fiber in static (batch) and continuous (column) photoreactors.

Figure 6a describes the MB rate removal by the PAN nanofiber and goethite nanoparticles as a reference test, under the same Bach reaction conditions. The results showed that within the first 15 min of the treatment, the removal rate of MB was 4.6% and 9.5%, then after half an hour, it was 4.8% and 10.1% for the PAN nanofiber and goethite nanoparticles, respectively, indicating that the adsorption–desorption equilibrium was almost reached within half an hour. After irradiation by visible light, the removal rates of MB via nanoparticles significantly increased and reached 95% after only 3 h. However, the PAN nanofiber did not show any significant increase after the first 30 min of the treatment. This proves, on one hand, the higher photo-reactivity of the generated goethite nanoparticles, and the inert photochemical shape of the fiber surface on the other hand, which indicated that the semiconductor photocatalytic reactions were the main pathway for MB degradation.

#### 3.2.2. Photocatalytic Decomposition over CNF

Investigating the capacity of CNF as a catalyst in the removal of pollutants starts by determining its adsorption capacity and the interactions between the adsorbate (pollutants) and the adsorbent (catalyst). Figure 6 b shows the low capacity, less than 5%, of the CNF in the adsorption of MB from water. The adsorption of MB on the surface of the CNF follows the Langmuir model, which assumes that the adsorption of MB occurs in a monolayer on the surface of the CNF (Table 3).

The photocatalytic degradation process is advanced than the adsorption process; photocatalysis not only aims to remove MB from the solution but also to mineralize this dye. Therefore, this section moves one step forward from analyzing the removal of MB from the solution by adsorption at the surface of CNF to the effect of the visible light exposure. The performance of the CNF as a photocatalyst for MB degradation under visible light (660 nm) is shown in Figure 6. The batch and continuous (column) photoreactor tests show comparable efficiency of the CNF to remove more than 90% MB solution after 5 h of visible light irradiation. It is worth noting that in the absence of CNF, no significant MB degradation was observed (result not shown). The MB degradation is basically based on the visible light excitation of the iron content of the CNF, which leads to the formation of electron-hole pairs (Equation (4)).

FeOOH + hν → FeOOH(e^−^, h^+^) (4)

The photocatalytic degradation of MB in the presence of -FeOOH may be explained by the generation of photoinduced charge carriers (electron-hole pairs) that decompose water molecules and generate very reactive oxidants, hydroxyl radicals (HO•) and other free radicals, namely, O_2_• and HO_2_• with high oxidative potential. The oxidative radicals formed due to the degradation of MB [39,40]. Furthermore, adsorption of MB on the surface of the CNF facilitates and speeds up the photodegradation of the adsorbed molecules in comparison with the molecules that are dissolved in the solutions. Indeed, the adsorbed MB molecules become close and in direct contact with the products of the photocatalytic reaction, i.e., free radicals at the surface of the CNF. The water molecules are oxidized at the CNF surface and produce free radicals that contribute to the photooxidation of MB. These results emphasize that the natural nano-goethite-based CNF structure is efficient as the nanowires with synthetic goethite of Londoño-Calderon [41] and Amani-Ghadim et al. [37], which photodegraded up to 90% of the dye in 2 h.

The photocatalytic degradation rate, 1.41 × 10^−2^ min^−1^, which is estimated based on Equation (3), is in agreement with the result obtained by Borker et al. [42] and Wu et al. [43] (1,47 × 10^−2^ min^−1^). The degradation rate 1.41 × 10^−2^ min^−1^ indicates fast degradation of MB at the surface of the CNF.

The reusability and the stability of the developed membrane composite are crucial characteristics for practical industrial and real-scale applications, taking into account the operational cost of the process.

**Figure 7:**
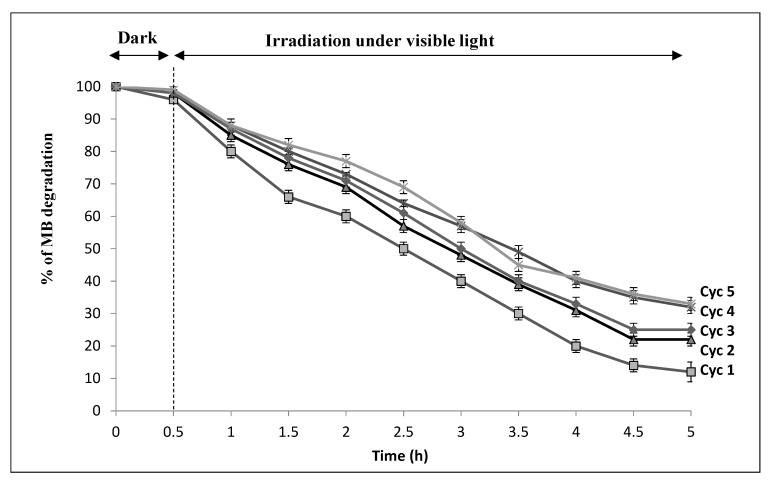
Recycling experiments for five cycles (Cyc) of PAN/goethite CNF for photocatalytic degradation of MB.

A reusability experiment for five cycles using the initial MB concentration is shown in Figure 7; during the first analysis, 97.5% of the MB elimination efficiency was reached within 2 h, and during the second analysis, the MB elimination was 88% within a longer period of 5 h. After five cycles, the photocatalytic efficiency of the CNF was still maintained at around 65%; this indicates that the PAN/goethite nano-composites exhibit good photocatalytic stability and higher reuse efficiency for the degradation of MB higher than five cycle.

**Figure 8:**
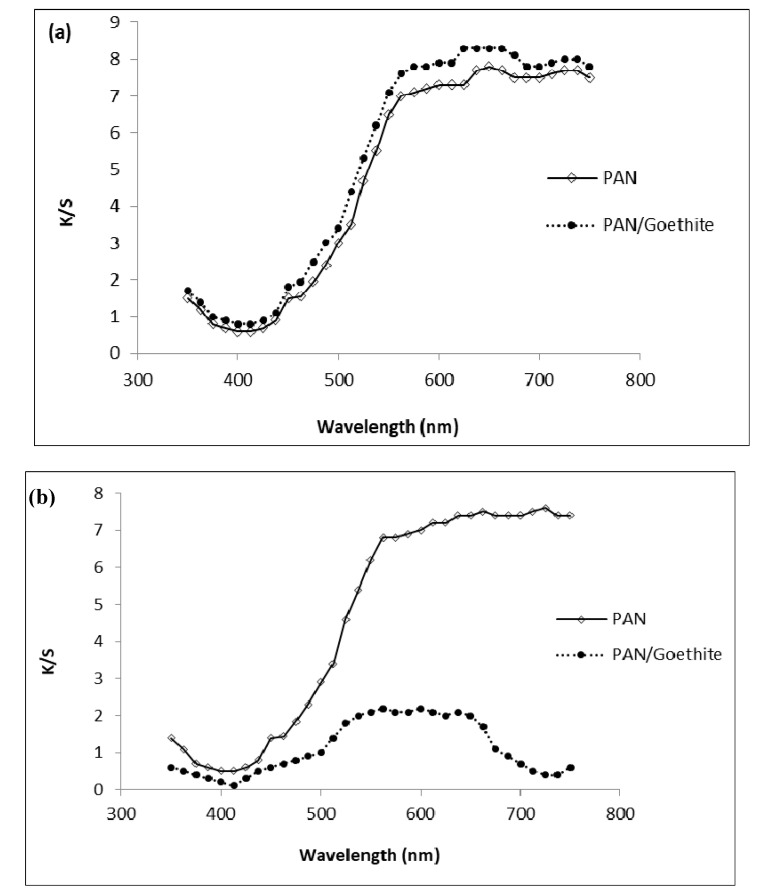
K/S curves of the PAN/goethite and the PAN before (**a**) and after (**b**) the photocatalytic process.

After the adsorption process, the nanofiber membranes became blue with different degrees of dye depth. The K/S value is usually used to evaluate the depth of dye adsorption [44], so it was employed here to measure the degree of MB adsorption and photocatalytic degradation. The corresponding K/S curves are shown in Figure 8a.

It can be seen that the respective K/S values of the PAN/goethite nanofiber were 8.40 and around 7.83 for the control (PAN nanofiber). The results indicated that the addition of goethite nanoparticles markedly increased the dye depth of the composite membranes. However, after irradiation, the membranes became light blue and the K/S values decreased strongly for the PAN/goethite nanofiber to 2.16 and weakly for the control nanofiber to 7.51, as shown in Figure 8b.

The preparation of the composite membrane as embedded by goethite nanoparticles and its use in MB adsorption and degradation could explain the treatment mechanism. During the adsorption process, MB molecules were adsorbed in the nanofibrous network, and some of the MB molecules were intercalated into the goethite pearl-like structure aggregates, lying parallel to the fiber layers.

When the membranes were exposed to visible light irradiation, the iron particles were continuously hydrolyzed and produced large quantities of oxidative groups such as O_2_^•−^, HO_2_^•^, and HO^•^, which accelerated the photooxidation of MB molecules. The nanocomposite membranes could be recycled, and the percentage of MB degradation exceeded 65% after five successive cycles.

Regarding the membrane stability during the treatment process, the concentration monitoring of ions that may be released from the fabricated CNF such as Fe, Al, and Si (natural goethite components) showed that there was no trace of those ions in the treated solution during the treatment, indicating relatively high stability of the fabricated CNF.

### 3.3. Bacteria Removal

**Figure 9:**
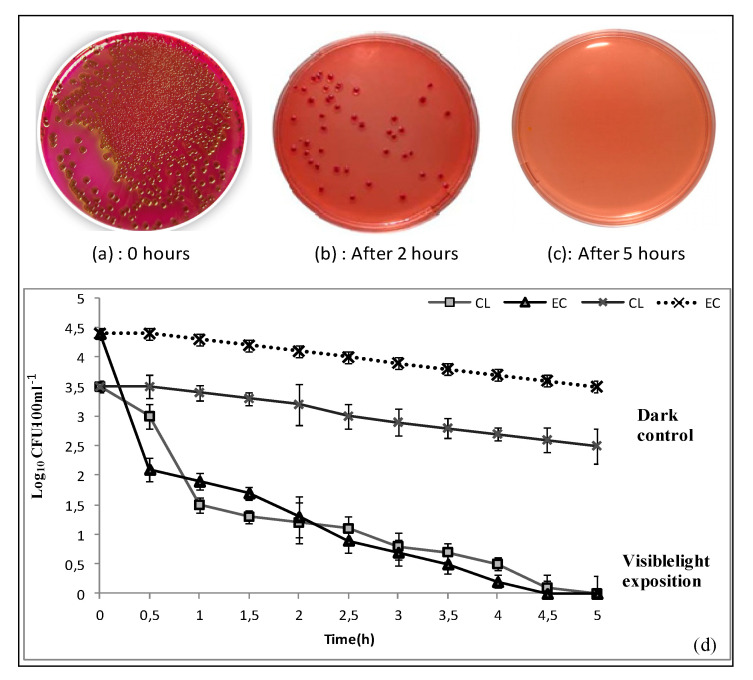
Images of *E. coli* colonies on a solid culture medium (**a**) before irradiation and after (**b**) 2 h and (**c**) 5 h irradiation in the presence of PAN/goethite nanofiber. (**d**) Evolution of bacterial inactivation by the natural nanocomposite fiber under continuous (column) treatment under visible light irradiation and in the dark (control). (Cl: *Clostridium perfringens*; EC: *E. coli*).

The results of the control experiment, i.e., in the dark, show the adsorption of *Clostridium perfringens* and *E. coli* to the CNF as shown in Figure 9. However, the bactericidal capacity of the irradiated CNF was much higher than its adsorption capacity. The bactericidal effect of CNF on the vegetative cells of *Clostridium perfringens* and *E. coli* is presented in Figure 9. The CNF photocatalytic efficiency achieved complete inactivation of 10^3^ CFU/100 mL of *Clostridium perfringens* spores (to the detection limit) in less than 5 h of irradiation and complete inactivation of 4.4 log (*E. coli)* in less than 4 h of irradiation. The inactivation of *E. coli* is one order of magnitude greater than that of *Clostridium perfringens*, mainly because *E. coli* is one of the most sensitive types of bacteria to disinfection as it has a vegetative cell form [3]. This is the reason why *E. coli* is described as an “easy target” for assessment of new/novel disinfection systems comparing to the bacterial spore’s form [8,11,45]. Irradiation of CNF excites its content of goethite that leads to reactions generating free oxygen radicals such as O^•^ and OH^•^. The free oxygen radicals are very strong oxidizing agents that are unselective and indiscriminately effective in the oxidation of organic pollutants and living organisms. The HO^•^ attacks the organisms’ DNA and other intracellular components that causes bacterial inhibition and death and thus enhances the disinfection process. Then, the CNF mechanism, deactivation of the tested organism, is based on the fact that goethite is a semiconductor with a band gap of 2.0–2.3 eV [46], which is typical for iron oxide. Irradiation of goethite generates oxygen-free radicals O_2_^•^ − and HO^•^ in several subsequent steps [47]. The free radicals generated by goethite activation can directly react with bacteria [47,48].

Accordingly, the photocatalytic degradation of dye and bacteria in the presence of FeOOH could be explained by the formation of photoinduced charge carriers (electron-hole pairs). The hydroxyl radicals are extremely strong, non-selective, and very reactive oxidants formed by the decomposition of water molecules in holes with high oxidative potential. The formation of hydroxyl radicals leads to the degradation of organic matter (dye and bacteria). Taking into consideration the photoinduced e^−^, h^+^ mechanism, the bandgap energy of synthesized rod-like-FeOOH nanoparticles was determined in previous work. The estimated bandgap of synthesized -FeOOH nanoparticles was determined to be approximately 2.5 EV, which is in good agreement with the literature.

The high photodegradation efficiency obtained in this study may be attributed to synergist and multiple effects of all metal oxides (Al_2_O_3_ and SiO_2_) that exist in the natural goethite sample, which positively affects the photocatalytic activity [49,50], but the greater role was played by iron due to its abundance, and the efficiency obtained under visible irradiation may be a promising key to enhance the solar disinfection process. Since the site of photocatalytic decomposition is physically separate from the reservoir containing contaminated water, the need for removal of the photocatalytic material is avoided.

All in all, the obtained results show that the goethite CNF exhibited high efficiency of bacteria removal compared to several other materials under visible light [51,52,53]. Moreover, the technology developed presents many advantages such as the natural resources of the used materials, which made the process low cost. Thus, the photocatalytic water treatment system described in this work can be easily modified for point-of-use applications.

## 4. Conclusions

In this work, a novel composite, flexible, and high-surface-area CNF was fabricated using electrospinning by embedding natural goethite nanoparticles in PAN. The adsorption and the photocatalytic activity of CNF were evaluated for both dye degradation and bacteria inactivation. The results demonstrated that the photoexcitation of the PAN/goethite CNF removed more than 90% of MB solution in 5 h under visible light radiation. In terms of reusability, it can be seen that the percentage of MB degradation always reached more than 65% after five successive cycles, indicating a promising reuse performance of the CNF in industrial applications. This performance was also confirmed by measurement of the K/S ratio as the relative color strength and staining, of which the result showed that the generated composite first adsorbed the dye and then degraded it through the photocatalysis process. The nanocomposite showed high efficiency (>90%) in the deactivation of *E. coli* and *Clostridium perfringens* in less than 5 h under visible light irradiation. In fact, the photoexcitation of the PAN/goethite CNF could remove about 4.4 and 3.5 log units of *E. coli* and *Clostridium perfringens*, respectively. Finally, one more potential prospect of this newly fabricated nanocomposite is the use in the decentralized or on-site treatment of water and wastewater photoactivated by solar irradiation.

## Figures and Tables

**Table 1:** Quantitative results of the weight and atomic concentration of selected elements on the basis of EDX presented in Figure 3.

Element	Weight %	Atomic %
C	1.05 ± 0.31	2.54
O	33.38 ± 0.49	60.90
Al	0.30 ± 0.08	0.32
Si	0.61 ± 0.09	0.64
S	0.70 ± 0.11	0.64
Cl	5.11 ± 0.18	4.21
Fe	58.85 ± 0.60	30.76
Totals	100.00	100.00

**Table 2:** Quantitative results of the weight and atomic concentration of selected elements on the basis of EDX presented in Figure 5.

Element	Weight %	Atomic %
O	25.21 ± 0.59	53.65
Al	0.35 ± 0.09	0.44
Si	0.58 ± 0.09	0.70
S	0.36 ± 0.10	0.38
Cl	1.60 ± 0.14	1.54
Fe	54.82 ± 1.06	33.42
Co	17.8 ± 1.15	9.86
Totals	100.00	100.00

**Table 3:** The values of Langmuir parameters obtained using a non-linear method for MB adsorption onto PAN/goethite CNF.

Langmuir Model	MB
*q_m_ (mg/g)*	2.6
*K_L_ (l/mg)*	0.05
*R* ^2^	0.989
